# TonEBP in dendritic cells mediates pro-inflammatory maturation and Th1/Th17 responses

**DOI:** 10.1038/s41419-020-2632-8

**Published:** 2020-06-04

**Authors:** Byeong Jin Ye, Hwan Hee Lee, Eun Jin Yoo, Chae Young Lee, Jun Ho Lee, Hyun Je Kang, Gyu Won Jeong, Hyun Park, Whaseon Lee-Kwon, Soo Youn Choi, Hyug Moo Kwon

**Affiliations:** School of Life Sciences, Ulsan National Institute of Science and Technology, Ulsan, 44919 Republic of Korea

**Keywords:** Cell biology, Immunology

## Abstract

Dendritic cells (DCs) are potent antigen-presenting cells that link the innate and adaptive immune responses; as such they play pivotal roles in initiation and progression of rheumatoid arthritis (RA). Here, we report that the tonicity-responsive enhancer-binding protein (TonEBP or NFAT5), a Rel family protein involved in the pathogenesis of autoimmune disease and inflammation, is required for maturation and function of DCs. Myeloid cell-specific TonEBP deletion reduces disease severity in a murine model of collagen-induced arthritis; it also inhibits maturation of DCs and differentiation of pathogenic Th1 and Th17 cells in vivo. Upon stimulation by TLR4, TonEBP promotes surface expression of major histocompatibility complex class II and co-stimulatory molecules via p38 mitogen-activated protein kinase. This is followed by DC-mediated differentiation of pro-inflammatory Th1 and Th17 cells. Taken together, these findings provide mechanistic basis for the pathogenic role of TonEBP in RA and possibly other autoimmune diseases.

## Introduction

Dendritic cells (DCs) are professional antigen-presenting cells (APCs) that are uniquely capable of priming naïve T-cells, although macrophages and B-cells are also able to process and present antigens to T-cells^1^. The maturation process, which upregulates expression of MHC class II and co-stimulatory molecules at the cell surface, is central to DC function^2^. Mature DCs migrate to draining lymph nodes (LNs) and promote differentiation of naïve T-cells into effector T-cells; they also activate various other immune cells depending on the stimuli that they sense in LNs^2^. Accordingly, evidence from clinical studies and experimental models implicates DCs in the pathogenesis of most autoimmune diseases, including multiple sclerosis, systemic lupus erythematosus, and rheumatoid arthritis (RA)^3^.

RA, one of the most prevalent autoimmune diseases in humans, is characterized by chronic inflammation and destruction of bone and cartilage within joints, leading ultimately to chronic pain, severe disability, and increased mortality^4^. The diagnosis and management of early arthritis are pivotal to prevent damage from becoming clinically significant^5^. Because dysregulation of immune cells plays a critical role in RA pathogenesis, its reversal is a major therapeutic goal^6,7^. Although effective therapies are available, a significant number of RA patients do not respond well^8^. Accordingly, better understanding of the causative and disease-contributing factors is needed. DCs are emerging as an important cell population that plays a role in RA pathogenesis^9–11^. DCs are involved in initiating the disease via production of cytokines and presentation of arthritogenic antigens, which together activate autoreactive Th1 and Th17 cells; it is the latter that actually cause the damage associated with RA^9,10^. In addition, DCs contribute to the marked increase in leukocyte infiltration into synovial tissue^11^. Data from human patients and murine models of early RA show that DC numbers in joint-draining LNs increase prior to visible histological changes, demonstrating the importance of DCs in initiating RA^12^. Furthermore, reduced numbers of circulating DCs in patients with RA correlate with an enriched synovial population of DCs possessing a high T-cell stimulatory capacity^13–15^. Thus, DCs may be central to both onset and progression of RA and could be a logical target for treatment.

Tonicity-responsive enhancer-binding protein (TonEBP), also known as nuclear factor of activated T-cells 5 (NFAT5), belongs to the Rel family of transcriptional factors, which includes nuclear factor-kB (NF-kB) and NFAT1-4^16,17^. TonEBP was identified initially as the central regulator of cellular responses to hypertonic stress^16,18,19^. Recent emerging studies show the role of TonEBP in the development and activation of immune cells, particularly T-cells and macrophages in osmostress-dependent and osmostress-independent contexts^20,21^. TonEBP induces activation of pathogenic Th17 cells^22,23^ and pro-inflammatory macrophages^24–26^. As a result, numerous studies in humans and mice show that increased expression of TonEBP contributes to inflammatory and autoimmune diseases^26–30^. Conversely, downregulation of TonEBP reduces inflammation, thereby helping to prevent these diseases^27–30^. Emerging data indicate that single-nucleotide polymorphisms in *TONEBP* are associated with inflammation^28^, diabetic nephropathy^28,31^ and risk of type 2 diabetes mellitus^32^ in various human cohorts suggesting that variations in the level of TonEBP expression affect disease susceptibility^33^.

TonEBP is highly expressed in macrophages obtained from the synovium of patients with RA than in normal macrophages from healthy individuals^27^. Global TonEBP haplo-insufficiency in a mouse model of RA markedly prevented pannus formation and cartilage destruction, which was related to the reduced survival and pro-inflammatory activation of macrophages^27,30^. While the role of TonEBP in macrophages is well-established, its role in DCs is unclear. Here, we examined the intrinsic role of TonEBP in the maturation and functioning of DCs in the context of inflammatory arthritis. Lack of TonEBP in myeloid cells, including DCs and macrophages, alleviated disease severity in mouse models of inflammatory arthritis, as well as inhibited maturation of DCs and differentiation of Th1 and Th17 cells in draining LNs and inflamed joints. Importantly, we found that TonEBP promotes maturation and inflammatory responses of DCs in response to toll-like receptor 4 (TLR4) stimulation, and then it induces differentiation of pro-inflammatory Th1 and Th17 cells via p38 mitogen-activated protein kinase (MAPK).

## Results

### TonEBP-deficient myeloid cells reduce the severity of arthritis in mouse models

The blockade of RA development in TonEBP-haplodeficient mice^27,30^ led us to examine the role of myeloid TonEBP in a mouse model of inflammatory arthritis based on myeloid-specific TonEBP knockout; these mice are referred to as *TonEBP*^*fl/fl*^ *LysM-cre* mice. First, we generated *TonEBP*^*fl/fl*^ *LysM-cre* mice using the Cre-lox system (*TonEBP*^*fl/fl*^; *lysozyme 2 promoter driven-Cre*). Floxed TonEBP mice that did not express Cre recombinase (*TonEBP*^*fl/f*^ alone) were used as a control. In myeloid lineage cells (peritoneal macrophages, and bone marrow-derived macrophages (BMDMs) and bone marrow-derived-dendritic cells (BMDCs)) TonEBP levels were dramatically reduced in the *TonEBP*^*fl/fl*^ *LysM-cre* mice compared to their *TonEBP*^*fl/fl*^ littermates (Supplementary Fig. 1a) confirming genetic deletion of *TONEBP*.

**Fig. 1:**
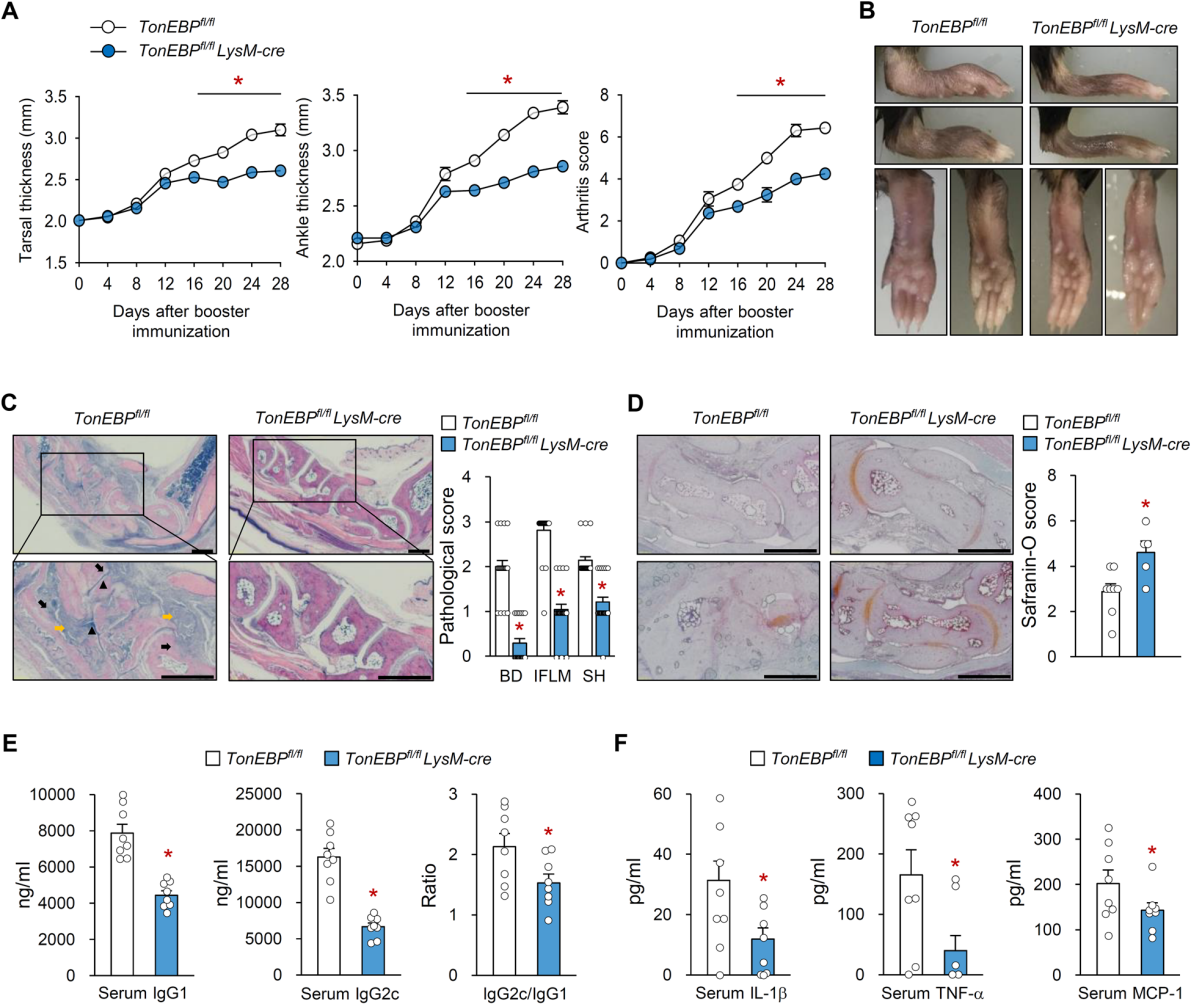
Myeloid TonEBP deficiency reduces the severity of collagen-induced arthritis. Collagen‐induced arthritis (CIA) was induced in male *TonEBP*^*fl/fl*^ *LysM-cre* mice (*n* = 24) and their *TonEBP*^*fl/fl*^ littermates (*n* = 21) aged 8–9 weeks as described in Supplementary Fig. 1a. **a** Tarsal thickness, ankle thickness, and arthritis scores were measured on the indicated days after booster immunization. **b** Representative images of hind paws on day 28 after booster immunization. **c** Representative images of ankle joint sections stained with haematoxylin and eosin (*left*), and pathological scores on day 28 after booster immunization (*right*). Black arrows indicate bone destruction (BD), yellow arrows indicate inflammatory cell infiltration (IFLM), and black arrowheads indicate synovial hyperplasia (SH). **d** Representative images (*left*) and quantification (*right*) of safranin-O staining (brown) of joint cartilages from *TonEBP*^*fl/fl*^ *LysM-cre* mice (*n* = 6) and their *TonEBP*^*fl/fl*^ littermates (*n* = 8). **e** Serum levels of IgG1 and IgG2c anti-collagen type II antibodies and IgG2c/IgG1 ratio on day 28 after booster immunization (*n* = 8). **f** Serum levels of IL-1β, TNF-α, and MCP-1 on day 28 after booster immunization (*n* = 8). *n* represents number of biologically independent animals. Scale bars, 500 μM. All data are expressed as mean ± s.e.m. *p < 0.05 vs. *TonEBP*^*fl/fl*^ (unpaired *t*-test).

We examined whether TonEBP expressed by myeloid cells plays a role in the collagen-induced arthritis (CIA) model, which is associated with autoimmunity to type II collagen. Mice were immunized with collagen, with a booster immunization given 14 days later (Supplementary Fig. 1b). Clinical signs of arthritis began to develop on Day 12 after the booster immunization and progressed to Day 28, the end of the experimental period (Fig. 1a, b). Disease severity in *TonEBP*^*fl/fl*^ *LysM-cre* mice was lower than that in control mice at Day 16 after boosting; this difference persisted up to Day 28, although arthritis onset was comparable in both groups of mice up to Day 12 (Fig. 1a, b). These clinical assessments were supported by histological examination of representative ankle joints. On Day 28, control ankle sections showed clear evidence of bone destruction, inflammatory cell infiltration, and synovial hyperplasia, all of which were markedly less severe in *TonEBP*^*LysM-KO*^ mice (Fig. 1c). Less cartilage damage was also observed in *TonEBP*^*fl/fl*^ *LysM-cre* mice (Fig. 1d). Next, we measured serum levels of anti-collagen II (CII) antibodies and inflammatory mediators (IL-1β, TNF-α, and MCP-1), which play an important role in the pathogenesis of CIA^10^. CII-specific IgG1 and IgG2c levels in *TonEBP*^*fl/fl*^ *LysM-cre* mice were markedly lower than those in control mice with CIA (Fig. 1e). Serum levels of IL-1β, TNF-α, and MCP-1 were also lower in *TonEBP*^*fl/fl*^ *LysM-cre* mice (Fig. 1f). We also examined the role of TonEBP in an adjuvant-induced arthritis (AIA) model. *TonEBP*^*fl/fl*^ *LysM-cre* mice and littermate control mice immunized with complete Freund’s adjuvant (CFA) development arthritis; progression was monitored by measuring paw volume for 14 days (Supplementary Fig. 1c). We noted a marked increase in the paw volume of control mice from 3 to 14 days post-CFA injection; however, the increase in hind paw volume of *TonEBP*^*fl/fl*^ *LysM-cre* mice was significantly lower than that in control mice (Supplementary Fig. 1d, e).

Taken together, these data demonstrate that the reduction of arthritis severity and inflammatory responses in *TonEBP*^*fl/fl*^ *LysM-cre* mice phenocopies those in global TonEBP-haplodeficient mice^27,30^, and that TonEBP in myeloid cells increases severity of arthritis.

### Deficiency of myeloid TonEBP inhibits immune responses in the paw tissue of CIA mice

**Fig. 2:**
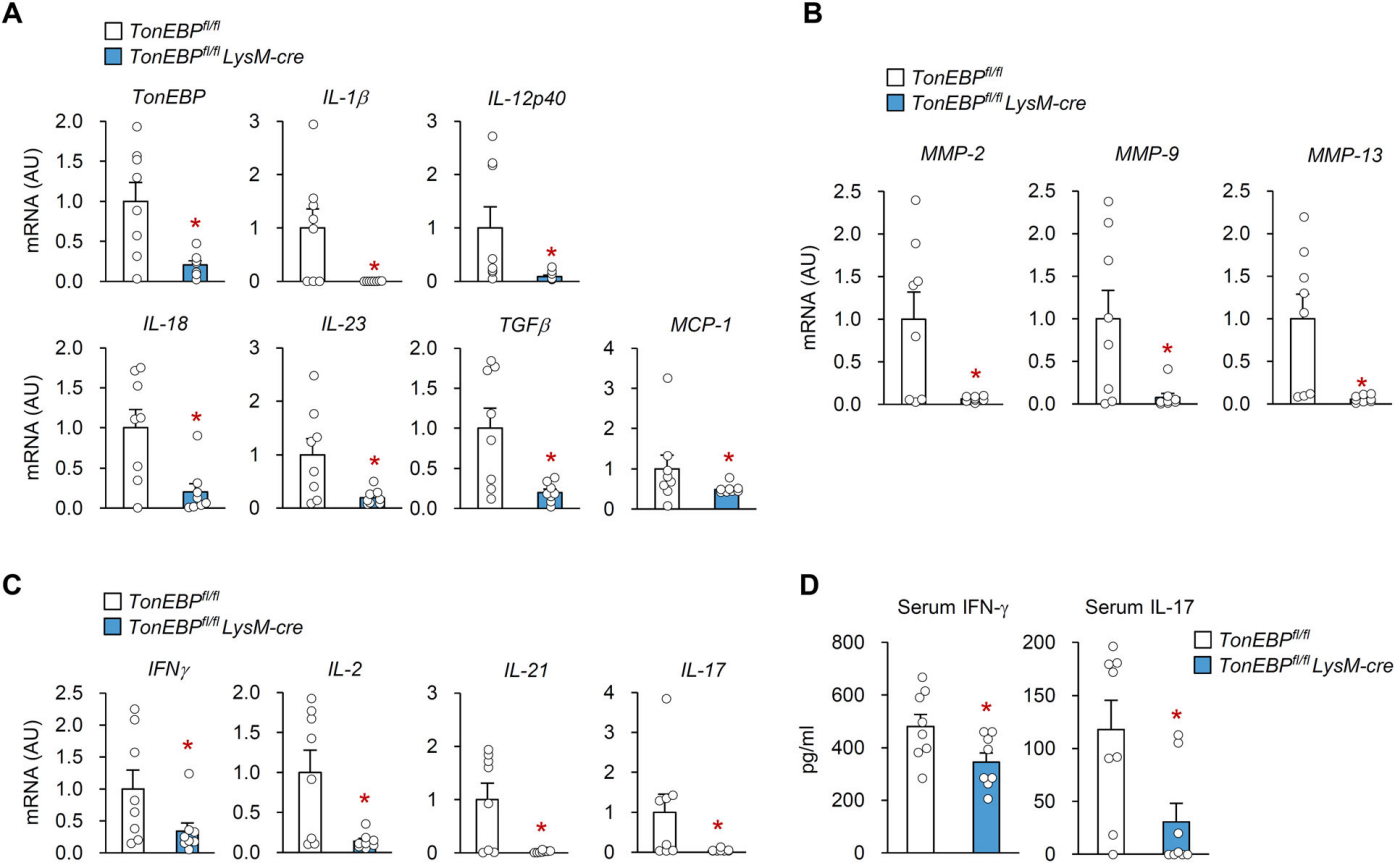
Myeloid TonEBP deficiency reduces immune responses in the paw tissues of CIA mice. CIA was induced as in Fig. 1 (*n* = 8). Expression of mRNA encoding TonEBP and pro-inflammatory cytokines **a**, matrix metalloproteinases **b**, and Th1 and Th17 cytokines **c** in paw tissue on day 28 after booster immunization was measured by quantitative RT-PCR. Relative expression of mRNA was normalized to that of the cyclophilin A gene and expressed as arbitrary units (AU). **d** Serum levels of IFN-γ and IL-17 on day 28 after booster immunization. *n* represents the number of biologically independent animals. All data are expressed as the mean ± s.e.m. *p < 0.05 vs. *TonEBP*^*fl/fl*^ (unpaired *t*-test).

Since *TonEBP*^*fl/fl*^ *LysM-cre* mice showed less severe inflammation and bone destruction (Fig. 1), we next examined RA-related immune responses in paw tissue. As the CIA model mimics many features of human RA and involves both the innate and adaptive immune systems, we performed the following experiments using the CIA model. First, we analyzed expression of mRNA encoding TonEBP in paw extracts from normal and CIA mice. The levels of TonEBP mRNA in the paw tissue of normal mice were below the limit of detection (Ct-value >40 in qPCR) and were higher in the paws of control mice with CIA (Supplementary Fig. 2). The levels in the paw tissue of *TonEBP*^*fl/fl*^ *LysM-cre* mice were lower than those in the control mice (Fig. 2a).

Next, we examined expression of mRNA encoding IL-1β, IL-12, IL-18, IL-23, TGF-β, and MCP-1, all of which are inflammatory cytokines relevant to RA pathogenesis, in paw tissue from mice with CIA. The paw tissue of normal mice exhibited undetectable levels of mRNA encoding any of these genes, whereas expression in CIA mice increased markedly (Supplementary Fig. 2a). Notably, expression of mRNA encoding these cytokines was markedly lower in *TonEBP*^*fl/fl*^ *LysM-cre* mice than in littermate controls (Fig. 2a). Consistent with the observed cartilage destruction (Fig. 1d), mRNA encoding matrix degrading enzymes (matrix metalloproteinase (MMP)-2, MMP-9, and MMP-13) was abundant in mice with CIA (Supplementary Fig. 2b) but much lower in *TonEBP*^*fl/fl*^ *LysM-cre* mice (Fig. 2b).

Given the importance of Th1 and Th17 cell activation in the pathogenesis of RA human patients and CIA mice^34,35^, we examined expression of mRNA encoding Th1 (IFN-γ and IL-2) and Th17 (IL-21 and Th17) cytokines, all of which have pro-inflammatory functions. These genes were highly expressed in the paw tissue of control CIA mice (Supplementary Fig. 2c) but much less so in the paw tissue of *TonEBP*^*fl/fl*^ *LysM-cre* mice (Fig. 2c). Consistent with these results, deletion of myeloid-specific TonEBP led to lower serum levels of IFN-γ and IL-17 than in control mice with CIA (Fig. 2d). Collectively, these data demonstrate a key role for myeloid TonEBP in CIA-induced paw inflammation and associated immune responses.

### Myeloid deficiency of TonEBP inhibits maturation of DCs and T-cell activation in CIA mice

**Fig. 3:**
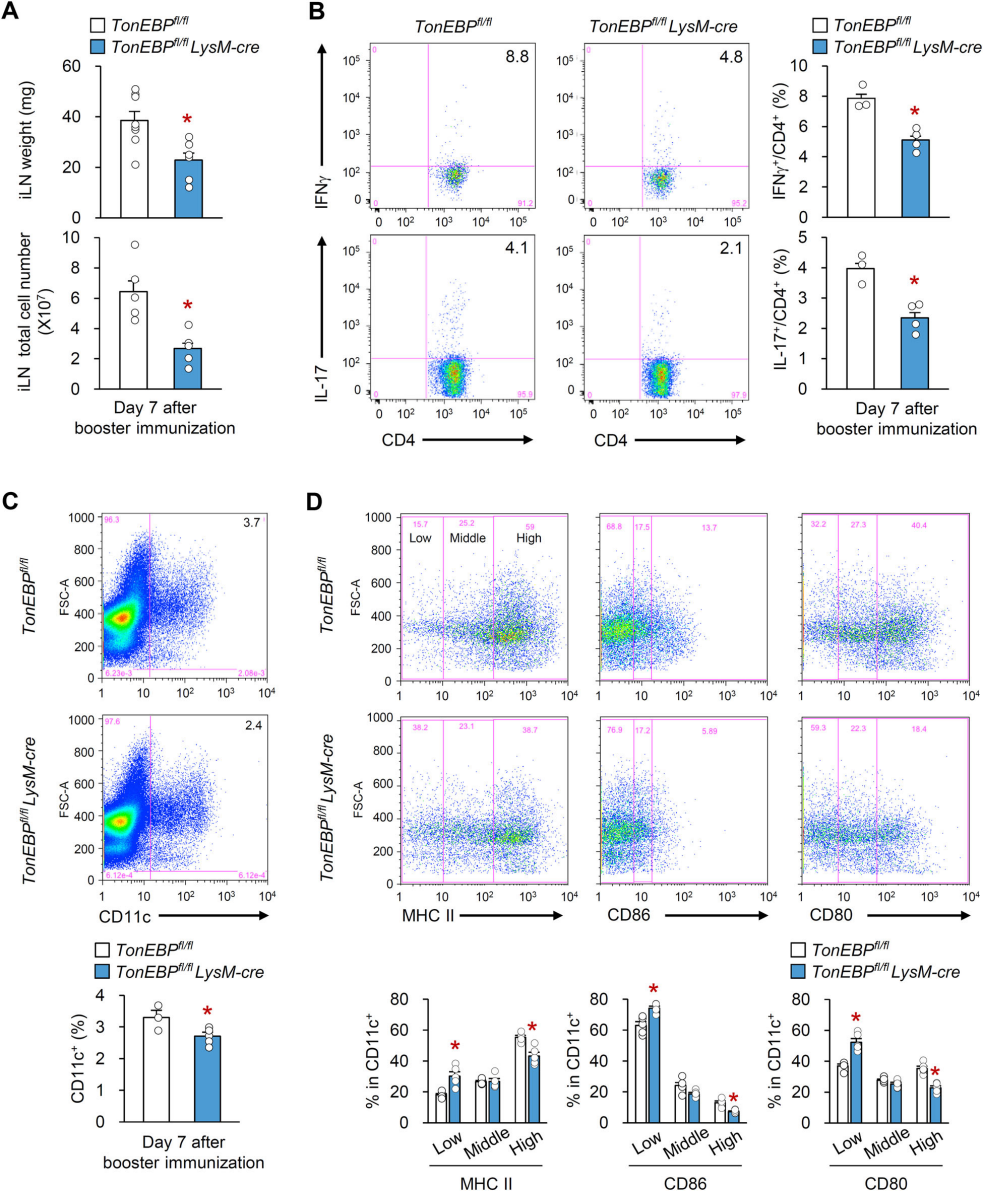
Myeloid TonEBP deficiency inhibits T-cell differentiation and maturation of DCs in CIA mice. CIA was induced as in Fig. 1. **a** Weight and total cell number in inguinal lymph nodes (iLNs) at day 7 after booster immunization (*TonEBP*^*fl/fl*^ *LysM-cre*, *n* = 6; *TonEBP*^*fl/fl*^, *n* = 8). **b** Representative flow cytometry plots (*left*) and quantification (*right*) of IFN-γ- and IL-17-expressing CD4^+^ T-cells from iLNs (*TonEBP*^*fl/fl*^ *LysM-cre*, *n* = 4; *TonEBP*^*fl/fl*^, *n* = 3). **c** Representative flow cytometry plots (*top*) and quantification (*bottom*) of CD11c^+^ dendritic cells within the cell population in iLNs (*TonEBP*^*fl/fl*^ *LysM-cre*, *n* = 4; *TonEBP*^*fl/fl*^, *n* = 3). **d** Representative flow cytometry plots (*top*) and quantification (*bottom*) of MHCII, CD86, and CD80 expression (low, middle, and high) on the surface of CD11c^+^ dendritic cells from iLNs (*n* = 5). *n* represents number of biologically independent samples. All data are expressed as the mean ± s.e.m. *p < 0.05 vs. *TonEBP*^*fl/fl*^ (**a**–**c**; unpaired *t*-test, **d**; one-way ANOVA).

Draining LN hypertrophy increased cellularity, and changes in the cellular composition of LNs are characteristics features of RA in humans and mice^36,37^. To assess whether TonEBP in myeloid cells affects hypertrophy of inguinal (i) LNs, we first examined the total cell number and weight of iLNs at Day 7 post-booster immunization with CII. iLNs from *TonEBP*^*fl/fl*^ *LysM-cre* mice weighed less and contained fewer cells than iLNs from control mice with CIA (Fig. 3a). Because *TonEBP*^*fl/fl*^ *LysM-cre* mice produced less Th1 and Th17 cytokines (Fig. 2c and d), we next examined CII-specific CD4^+^ T-cell responses in iLNs. Flow cytometry analysis revealed lower numbers of Th1 and Th17 cells in iLNs from *TonEBP*^*fl/fl*^ *LysM-cre* than those from control CIA mice (Fig. 3b and Supplementary Fig. 3a).

The maturation of DCs is the most important step in the trafficking of DC to the LNs^38^ and activation of naïve T-cells^39^ during RA development. Therefore, we examined the effect of myeloid TonEBP depletion on the distribution of DCs in iLNs. iLNs from *TonEBP*^*fl/fl*^ *LysM-cre* mice contained fewer DCs than those from control CIA mice at Day 7 post-booster immunization (Fig. 3c and Supplementary Fig. 3b). Furthermore, DCs from the iLNs of *TonEBP*^*fl/fl*^ *LysM-cre* expressed lower levels of MHCII and co-stimulatory molecules (CD80 and CD86), which are the hallmark of DC maturation (Fig. 3d). Given the reduced numbers of DCs in iLNs from *TonEBP*^*fl/fl*^ *LysM-cre* mice (Fig. 3c), we also examined the gene expression of CC-chemokine receptor 7 (CCR7), which have an important role in trafficking of DC to LNs, and its ligands CC-chemokine ligand 19 (CCL19) and CCL21^40,41^. There were no differences in the level of mRNA expression of these genes (Supplementary Fig. 3c) demonstrating that the myeloid TonEBP does not modulate the expression of CCR7, CCL19, and CCL21 in iLNs of mice with CIA.

Collectively these data suggest that myeloid TonEBP promotes maturation of DCs and activates T-cells in draining LNs of mice with CIA.

### TonEBP promotes DC maturation and inflammatory responses

We next addressed the intrinsic role of TonEBP during DC maturation. To do this, we generated BMDCs by culturing BM from *TonEBP*^*fl/fl*^ *LysM-cre* mice and their *TonEBP*^*fl/fl*^ littermates with granulocyte/macrophage colony-stimulating factor (GM-CSF). On day 6 of culture with GM-CSF, non-adherent and loosely adherent cells were transferred to Petri dishes to remove BMDMs^42,43^. After 1 day of culture, non-adherent cells and adherent cells were harvested separately. The non-adherent cells expressed higher levels of CD11c, MHCII, and co-stimulatory molecules (CD86 and CD80) but lower levels of F4/80 compared to adherent cells (Supplementary Fig. 4a). Furthermore, the levels of DC markers RelB (a transcription factor of DC), CCR7, CD86, and CD80 were significantly higher in non-adherent cells than adherent cells (Supplementary Fig. 4b). In contrast, expression of macrophage markers MafA and MafB was lower in the non-adherent cells (Supplementary Fig. 4c). Thus, the non-adherent cells were highly enriched for DCs.

**Fig. 4:**
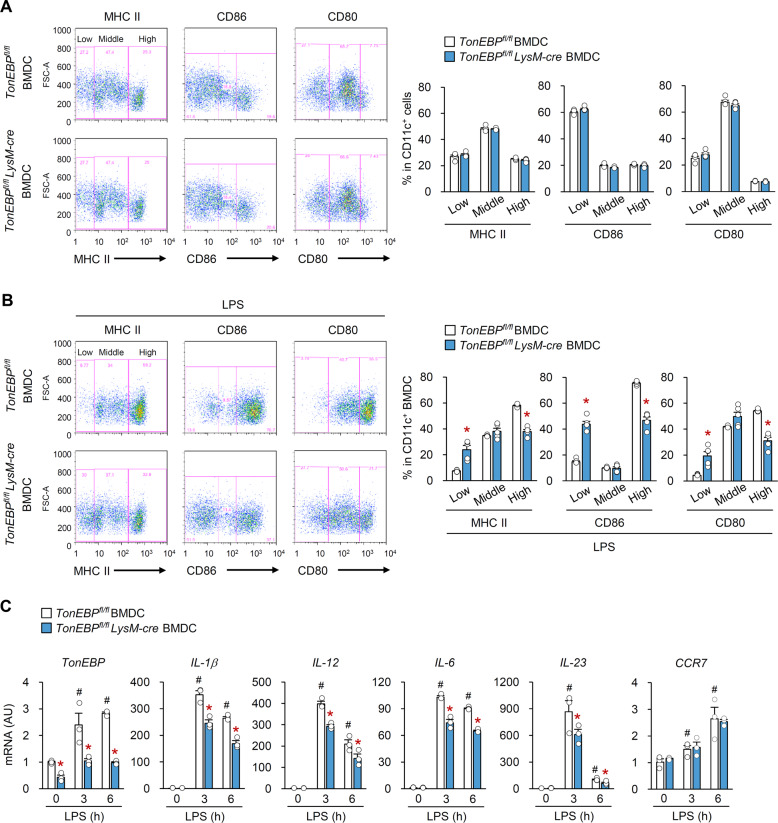
TonEBP is required for maturation and inflammatory responses of BMDCs in response to LPS. Bone marrow-derived dendritic cells (BMDCs) were generated from *TonEBP*^*fl/fl*^ *LysM-cre* mice and their *TonEBP*^*fl/fl*^ littermates. **a** The cell surface markers of DC maturation (MHCII, CD86, and CD80) were analyzed by flow cytometry. Representative flow cytometry plots (*left*) and quantification (*right*) for cell surface expression of MHCII, CD86, and CD80 (*n* = 4). **b** and **c** BMDCs were stimulated with LPS for 0, 3, 6, or 24 h. **b** Cell surface expression of MHCII, CD86, and CD80 was analyzed by flow cytometry after LPS stimulation for 24 h (*n* = 5). Representative flow cytometry plots (*left*) and quantification (*right*) for surface expression of MHCII, CD86, and CD80. **c** Expression of mRNA encoding pro-inflammatory cytokines and CCR7 was measured by quantitative RT-PCR after stimulation with LPS for 0, 3, or 6 h (*n* = 3). Data are expressed as arbitrary units (AU). *n* represents the number of biologically independent samples. All data are expressed as the mean ± s.d. #*p* < 0.05 vs. 0 h LPS. *p < 0.05 vs. *TonEBP*^*fl/fl*^ BMDC (one-way ANOVA).

We first examined the effect of TonEBP on development of DC from BM. Notably, GM-CSF cultures of BM of *TonEBP*^*fl/fl*^ *LysM-cre* and control mice had similar number of BMDC and comparable percentage of CD11c expression (Supplementary Fig. 4d). Furthermore, TonEBP-deficient CD11c^+^ BMDCs displayed similar levels of MHCII, CD86, and CD80 under steady-state conditions (Fig. 4a). Similarly, expression of mRNA encoding MHCII-related genes (H2-Aa, H2-Ab, and CD74) in BMDCs was not affected by lack of TonEBP; however, TonEBP-deficient BMDMs showed reduced expression of mRNA encoding CD74, H2-Aa, and H2-Ab, a finding in line with a previous report (Supplementary Fig. 4e)^44^. These data suggest that lack of TonEBP does not affect DC development and homeostasis.

Next, to address whether TonEBP is involved in DC maturation and inflammatory responses, we stimulated BMDCs with the TLR4 ligand, lipopolysaccharide (LPS), which is a potent inducer of DC maturation and inflammatory responses^45^. Upon stimulation with LPS for 24 h, the percentage of cells expressing high levels of MHCII, CD86, and CD80 protein increased, whereas that of cells expressing low and intermediate levels decreased (Supplementary Fig. 5a). Notably, fewer BMDCs from *TonEBP*^*fl/fl*^ *LysM-cre* mice expressed high levels of these proteins relative to BMDCs from control mice, whereas the percentage of cells expressing low levels of these proteins was higher than that in control mice (Fig. 4b). The lack of TonEBP expression in BMDCs did not affect expression of gene encoding TLR4 and CD14, a co-receptor for TLR4 activation (Supplementary Fig. 5b). In addition, cell viability and apoptosis in BMDCs from *TonEBP*^*fl/fl*^ *LysM-cre* mice were comparable to those in control mice under basal and LPS-stimulated conditions (Supplementary Fig. 5c, d). These data suggest that TonEBP plays a critical role in the maturation of BMDC in response to LPS.

We also examined expression of pro-inflammatory cytokines by LPS-stimulated BMDCs. Expression of TonEBP mRNA and protein increased after LPS stimulation; however, a significant reduction was observed in TonEBP-deficient BMDCs (Fig. 4c). As expected, LPS-treated control BMDCs expressed high levels of pro-inflammatory cytokines IL-1β, IL-12, IL-6, and IL-23; however, levels were lower in TonEBP-deficient BMDCs (Fig. 4c). On the other hand, expression of mRNA encoding CCR7 was not affected by TonEBP deficiency (Fig. 4c) in line with results from iLNs (Supplementary Fig. 3c).

Collectively, these data demonstrate that TonEBP promotes maturation and inflammatory responses by BMDCs in response to LPS.

### TonEBP in DCs promotes proliferation of CD4^+^ T-cells and subsequent differentiation into Th1/Th17 cells

**Fig. 5:**
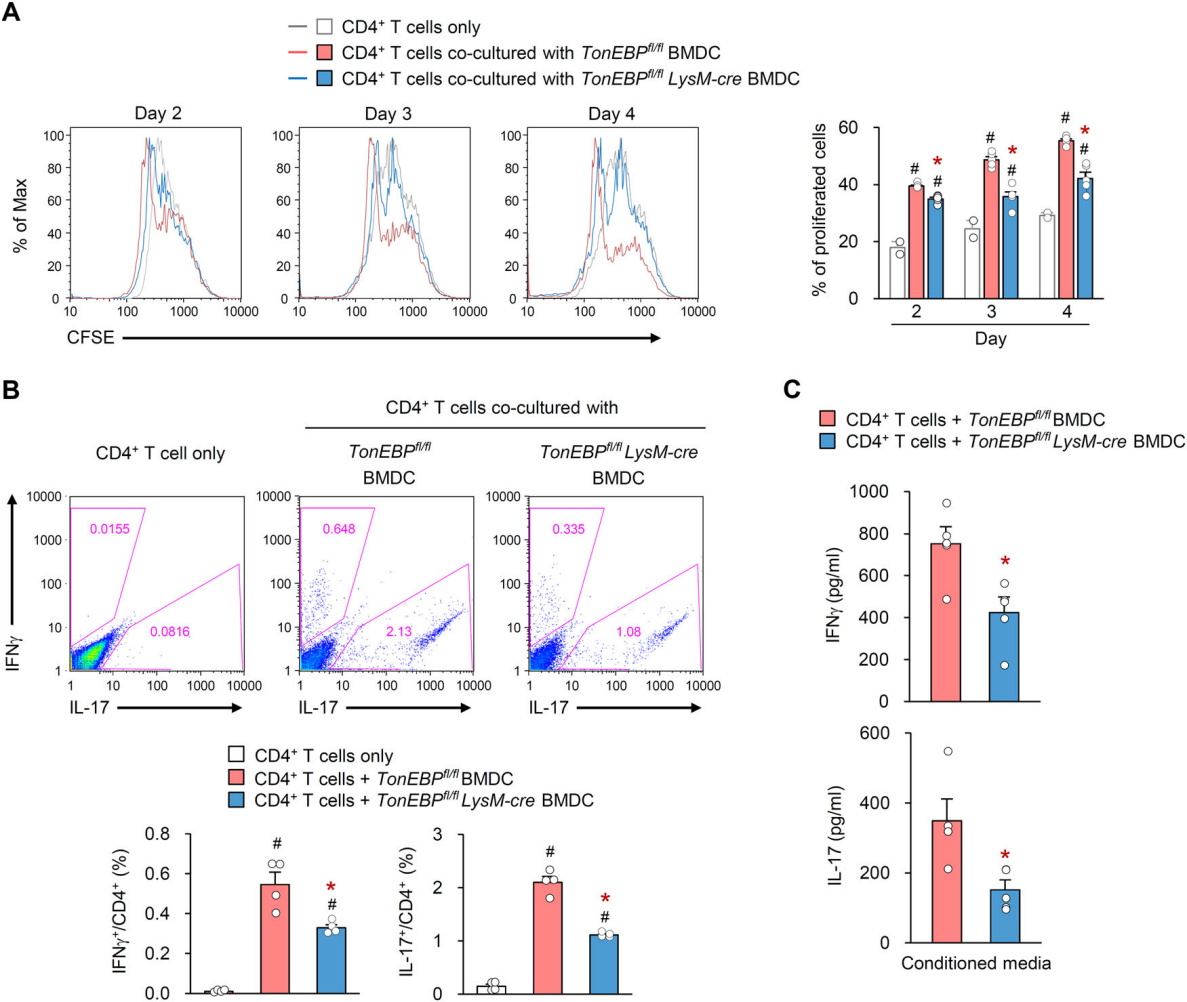
TonEBP in DCs promotes proliferation of CD4^+^ T-cells and subsequent differentiation into Th1/Th17 cells. CD4^+^ T-cells isolated from CII-immunized wild-type mice were co-cultured with LPS/CII-stimulated BMDCs isolated from *TonEBP*^*fl/fl*^ *LysM-cre* mice and *TonEBP*^*fl/fl*^ littermates. CD4^+^ T-cells alone without BMDCs were used as a control. **a** Proliferation of CD4^+^ T-cells was assessed based on reduction of fluorescence of 5(6)-carboxyfluorescein diacetate succinimidyl ester (CFSE) at 2, 3, or 4 days (*n* = 5). Representative flow cytometry plots (*left*) and quantification (*right*) of the percentage of proliferated CD4^+^ T-cells. **b** Representative flow cytometry plots (*top*) and quantification (*bottom*) of the percentage of IFNγ^+^ and IL-17^+^ cells within the CD4^+^ T-cell population (*n* = 4). **c** Concentrations of IFNγ and IL-17 in conditioned medium from CD4^+^ T-cells co-cultured with LPS/CII-stimulated BMDCs (*n* = 5). *n* represents the number of biologically independent samples. All data are expressed as the mean ± s.d. #*p* < 0.05 vs. CD4^+^ T-cells only. *p < 0.05 vs. *TonEBP*^*fl/fl*^ BMDC (unpaired *t*-test).

Next, we asked whether TonEBP in DCs is responsible for differentiation of Th1 and Th17 cells. We isolated CD4^+^ T-cells from CII-immunized wild-type mice and co-cultured them with LPS plus CII-stimulated BMDCs isolated from *TonEBP* ^*fl/fl*^ or *TonEBP*^*fl/fl*^ *LysM-cre* mice for 4 days. We examined proliferation of CD4^+^ T-cells and found that cells co-cultured with TonEBP-deficient BMDCs proliferated markedly less well than those from control BMDC at 2–4 days (Fig. 5a). In addition, the percentages of IFNγ^+^ and IL-17^+^ cells within the CD4^+^ T-cells population co-cultured with TonEBP-deficient BMDCs were reduced (Fig. 5b). Consistent with this, CD4^+^ T-cells co-cultured with TonEBP-deficient BMDCs secreted less IFNγ and IL-17 (Fig. 5c). These data suggest that TonEBP in DCs is required to induce CD4^+^ T-cell proliferation and differentiation into Th1/Th17 cells.

### TonEBP in DCs regulates LPS-mediated DC maturation via p38 MAPK

**Fig. 6:**
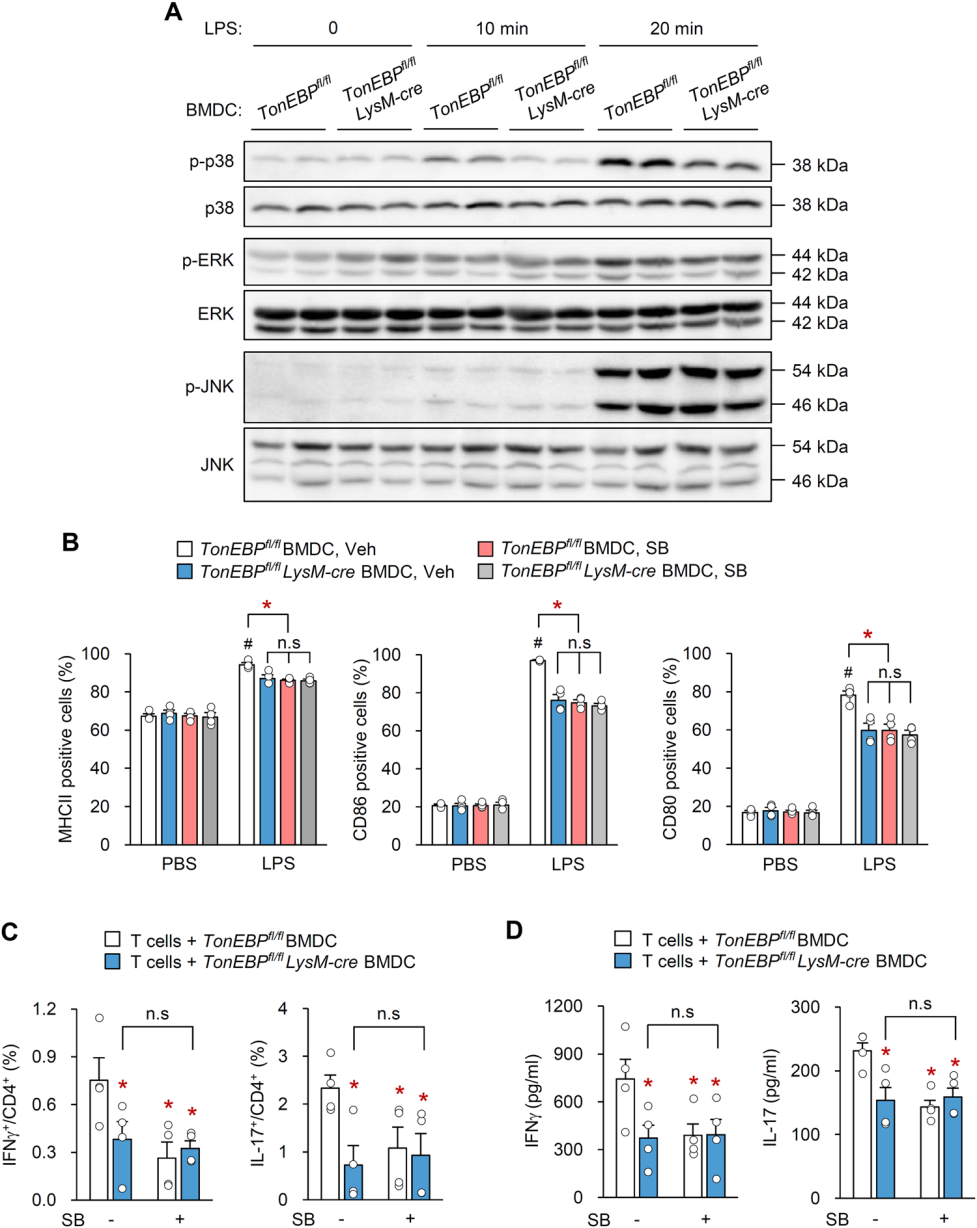
TonEBP in DCs controls LPS-mediated DC maturation via p38 MAPK. BMDCs were generated from *TonEBP*^*fl/fl*^ *LysM-cre* mice and *TonEBP*^*fl/fl*^ littermates followed by stimulation with LPS for 0, 10, 20 min, or 24 h. **a** Representative immunoblots showing activation of p38, ERK, and JNK MAPKs after 0, 10, or 20 min of LPS-stimulation. **b** BMDCs were pretreated with SB203580 (SB), an inhibitor of p38 MAPK, followed by LPS stimulation for 24 h. Cell surface expression MHCII, CD86, and CD80 was analyzed by flow cytometry. **c** and **d** BMDCs were incubated with LPS/CII or LPS/CII/SB for 24 h followed by co-culture with CD4^+^ T cells for 3 days. **c** The percentages of IFNγ^+^ and IL-17^+^ cells within the CD4^+^ T cell population (*n* = 4). **d** Levels of IFNγ and IL-17 protein in culture medium from CD4^+^ T cells co-cultured with BMDCs (*n* = 4). *n* represents the number of biologically independent samples. All data are expressed as the mean ± s.d. #*p* < 0.05 vs. PBS. *p < 0.05 vs*. TonEBP*^*fl/fl*^ BMDC, Veh **b** or *TonEBP*^*fl/fl*^ BMDC **c** (one-way ANOVA).

Next, we examined potential pathways that mediate TonEBP function during maturation of BMDCs in response to LPS. Because lack of TonEBP expression by BMDCs attenuated surface expression of maturation-related molecules in response to LPS (Fig. 4b), we first examined expression of genes encoding MHC II. TonEBP-deficient DCs did not show altered expression of genes encoding MHC II proteins or its transcriptional coactivator class II transactivator (CIITA) regardless of LPS treatment (Supplementary Fig. 6a); by contrast, their expression in TonEBP-deficient BMDMs decreased (Supplementary Fig. 6b), as previously reported^42^. This indicates that TonEBP affects cell surface expression of DC maturation markers in response to LPS without changes in the level of expression. Because the surface expression is dependent on MAPKs^46,47^, we next assessed whether TonEBP modulates activation of MAPKs (ERKs, JNKs, and p38) in DCs stimulated by LPS. As expected, LPS activated all MAPKs in control BMDCs (Fig. 6a). Interestingly, phosphorylation of p38 (but not ERKs or JNKs) in TonEBP-deficient cells was lower than that in control BMDCs (Fig. 6a), suggesting that TonEBP plays a role in p38 MAPK activation.

To test whether TonEBP-mediated DC maturation is dependent on the p38 MAPK pathway, we pretreated control and TonEBP-deficient BMDCs with SB203580, an inhibitor of p38 MAPK, and then stimulated them with LPS. Surface expression of MHCII, CD86, and CD80 in response to LPS was reduced markedly by TonEBP deficiency or addition of SB203580 (Fig. 6b). Notably, inhibition of p38 MAPK had no additional effect on reduced surface expression of MHCII, CD86, and CD80 induced by TonEBP deficiency (Fig. 6b). Neither TonEBP deficiency nor SB203580 affected the surface expression of MHCII, CD86, and CD80 by cells treated with PBS (Fig. 6b). Furthermore, to examine the effect of SB203580 on TonEBP-mediated differentiation of CD4^+^ T-cells, we incubated BMDCs from *TonEBP* ^*fl/fl*^ or *TonEBP*^*fl/fl*^ *LysM-cre* mice for 24 h with LPS plus CII, or with LPS, CII, and SB203580, and then co-cultured them with CD4^+^ T-cells for 3 days. SB203580 had no additional effect on differentiation into Th1 and Th17 cells (Fig. 6c) or on secretion of IFN-γ and IL-17 (Fig. 6d) over those observed in CD4^+^ T-cells co-cultured with TonEBP-deficient BMDCs. Collectively, these data demonstrate that TonEBP promotes activation of DCs in response to LPS by activating p38 MAPK.

## Discussion

**Fig. 7:**
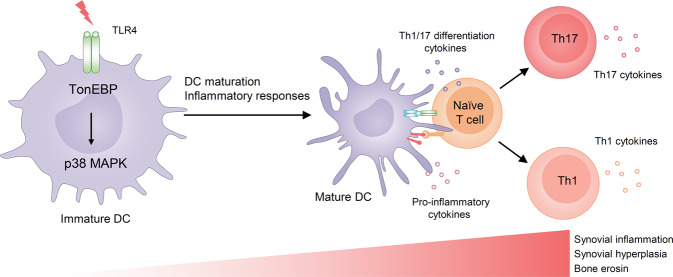
TonEBP expression by DCs promotes maturation and contributes to development of rheumatoid arthritis. TonEBP expression by dendritic cells (DCs) promotes maturation and inflammatory responses in response to TLR4 stimulation via activation of p38 MAPK. Maturation of DCs stimulates differentiation of CD4^+^ naïve T-cells into Th1 and Th17 cells, leading to the development of rheumatoid arthritis.

Previously, we reported that global TonEBP haplo-insufficiency protected mice against RA, and that this was related to reduced survival and activation of macrophages^27,30^. Here, we used myeloid-specific TonEBP-deficient mice to show that TonEBP expressed by myeloid cells, including macrophages and DCs, contributes to progression of RA. Moreover, we reveal the intrinsic functions of TonEBP during DC maturation and inflammatory responses. TonEBP expressed by DCs promotes TLR4-mediated maturation via p38 MAPK and, as a result, increases pro-inflammatory CD4 T-cell responses (Fig. 7).

A role for TonEBP in DCs is suggested by the crucial role played by DCs during pathogenesis of most autoimmune diseases, including RA^3^. Autoimmune diseases are caused by disruption of immune tolerance to self-antigens, resulting in damage to (or dysfunction of) normal tissues. The dysregulated activity of DCs breaks self-tolerance; thus DCs are an attractive therapeutic target. Indeed, some treatments for autoimmune diseases target DCs. TNF-α blockade, an effective therapy for RA, acts by impairing the survival and function of DCs^48^. Therapeutic vaccination with IL-27-conditioned DCs suppresses the development of experimental autoimmune encephalomyelitis by reducing pathogenic T-cell responses^49^. IFN-β, a first-line disease-modifying therapy for multiple sclerosis, reduces migration of DCs to the draining LNs and reduced their capacity to activate CD4^+^ effector T-cells^50^. Therefore, identification of TonEBP as a novel regulator of DC maturation and function provides new insight into the mechanisms underlying the development of autoimmune diseases, and may guide possible therapeutic approaches. More importantly, our finding that DCs lacking TonEBP express normal levels of MHC class II and co-stimulatory molecules (Fig. 4a) is of great interest because DCs also play a key role in maintaining immune tolerance under steady-state conditions, i.e., in the absence of overt infection or inflammation^51^. Thus, this study provides an opportunity to further our understanding of the role of DC-intrinsic TonEBP in immune tolerance and autoimmunity.

Based on several lines of evidence from the present and previous works^27,28,30^, we identified TonEBP-dependent cellular immune responses that are potential new targets for therapeutic intervention in RA. First, genetic variants of TonEBP in a cohort of healthy humans are associated with expression of systemic inflammatory factors, including serum MMP and IL-1β^28^. Importantly, these genes increase the risk of RA in humans^4^. Our present and previous studies show that TonEBP deficiency reduces the severity of arthritis in mouse models due to reduced expression of inflammatory factors^27,30^, suggesting that genetic variability in expression of TonEBP leads to differential susceptibility for RA. Second, TonEBP-deficient DCs (in this study) and macrophages^44^ attenuate activation of pro-inflammatory effector T-cells, the causative agents of RA. Third, many TonEBP-dependent genes in DCs and macrophages are linked functionally to human arthritis or experimental arthritis. Taken together, these data suggest that targeting TonEBP may be of benefit to individuals with arthritis.

DCs and macrophages play important roles in the pathogenesis of RA^52,53^. DCs and macrophages arise from a common progenitor and share a variety of roles, including antigen presentation, participation in T-cell homeostasis, and maintenance of immunity, while at the same time maintaining distinct functions within the immune system and in the context of disease^3,54,55^. MHC class II molecules are constitutively expressed on the cell surface of DCs and macrophages, and expression increases in response to cell-specific stimuli. Thus, these cells present antigens to CD4^+^ T-cells^3,56^. Interestingly, the present and previous findings demonstrate that DCs and macrophages differ with respect to their dependence on TonEBP for MHC class II expression. Here, we found that TonEBP deficiency did not affect constitutive expression of MHC class II molecules by DCs under basal conditions (Fig. 4a); however, it did inhibit LPS-inducible expression of MHC II (Fig. 4b). By contrast, we and others^44^ showed that macrophages lacking TonEBP show reduced basal and IFN-γ-induced expression of mRNA encoding MHC class II, as well as surface expression of the protein. Furthermore, TonEBP expression by DCs promotes cell surface expression of MHC class II and co-stimulatory molecules in response to LPS via p38 MAPK. This finding is interesting because TonEBP in macrophages did not affect activation of p38 MAPK in response to LPS^25^. These data suggest that TonEBP constitutes a cell-specific mechanism for pro-inflammatory activation of DCs.

In summary, our results reveal that the essential nature of TonEBP expression by DCs is to induce differentiation of effector T-cells. These findings have therapeutic implications in that targeting TonEBP in DCs may be an approach to treating disorders of the immune system and immune-associated diseases.

## Materials and methods

### Mice

All studies used male C57BL/6J mice. Mice carrying the loxP-targeted TonEBP gene (*TonEBP*^*fl/fl*^) have been described^57^ and were provided by Dr. Neuhofer. Lysozyme 2-cre knock-in mice, known as LysM-cre mice, were obtained from Jackson Laboratories (Bar Harbor, ME, USA). *TonEBP*^*fl/fl*^ mice were crossed to LysM-cre mice to generate mice lacking *TonEBP* in myeloid cells. Age-matched and sex-matched littermate control animals were used for all experiments. Animals were randomly assigned to experimental groups based on body weight. Blinding was not used. All animal procedures were approved by and performed according to guidelines of the Institutional Animal Care and Use Committee of the Ulsan National Institute of Science and Technology (UNISTACUC-16-08).

### Collagen-induced arthritis

Male *TonEBP*^*fl/fl*^ LysM-cre mice and littermate control *TonEBP*^*fl/fl*^ mice (aged 8–9 weeks) were immunized by intradermal injection (at the tail base) of 100 μg chicken type II collagen (CII; Chondrex, Redmond, WA, USA) emulsified in CFA containing 100 μg of heat-killed *Mycobacterium tuberculosis* H-37RA (Chondrex). Mice received a booster immunization of CII in incomplete Freund’s adjuvant (IFA; Chondrex) into the left hind paw on Day 14. Paw swelling was measured at the indicated times using a caliper. On Day 28 after booster immunization, arthritis scores were graded 0–4 (1) 0 = normal; 1 = mild inflammation of a single area (i.e., midfoot, ankle, or toes); 2 = moderately severe arthritis involving toes and ankle or midfoot; 3 = severe arthritis involving the entire paw; and 4 = severe arthritis resulting in ankylosis and loss of joint movement. Scores were assigned by an investigator blinded to the mouse genotype. The hind paw in which the booster immunization had been administered was excluded from the evaluation. The right hind paw and forepaw were assessed; thus the maximum possible arthritis score was 8^58^. No statistical methods were used to pre-determine sample sizes, but the sample sizes are comparable to those in previous studies^27,30^.

### Histology

At Day 28 post-booster immunization, mice were sacrificed prior to histological analysis. Fore and hind limbs that received no booster immunization were fixed in 10% formalin, decalcified, dehydrated, and then embedded in paraffin. Paraffin sections were stained with hematoxylin and eosin (Sigma-Aldrich, Saint Louis, MO, USA) for morphological evaluation, or stained with safranin-O (Sigma-Aldrich) to examine glycosaminoglycan distribution in cartilage. Histological observation and pathologic scoring were performed by observation under a light microscope (X51; Olympus, Tokyo, Japan). The severity of three parameters of arthritis (bone erosion, joint infiltration by inflammatory cells, and synovial hyperplasia) was scored on a scale from 0 to 3 (0, absent; 1, weak; 2, moderate; and 3, severe)^59^. For gene expression analysis (see below for details), paws were snap frozen in liquid nitrogen at the indicated times and subjected to mechanical disruption using a Polytron homogenizer (Kinematica AG, Luzern, Switzerland).

### Cell preparation and culture

Single-cell solutions were prepared from iLNs draining the paw by digestion with collagenase (1 mg/ml) and DNase I (0.1 mg/ml)^60^. The total number of cells was determined by microscopic observation in a hemocytometer after trypan blue-staining. For cytokine detection, cell suspensions derived from iLNs were plated in 96-well plates and then stimulated for 5 h with cell activation cocktail containing 50 ng/ml phorbol 12-myristate 13-acetate (Sigma-Aldrich) and 1 µg/ml ionomycin (Sigma-Aldrich), followed by stimulation for 12 h with GolgiStop^TM^ (BD Biosciences, San Jose, CA, USA). DCs and macrophages were generated from murine BM. Briefly, BM was flushed from the femurs of *TonEBP*^*fl/fl*^ LysM-cre mice and littermate control *TonEBP*^*fl/fl*^ mice. A single-cell suspension was obtained by passing the BM through a 25 gauge needle and filtering through a cell strainer (70 μm). The preparation was then divided into two separate cultures. BMDCs were generated as described^43,61^ with slight modifications. BM were cultured in RPMI 1640 medium (Lonza, Cologne, Germany) supplemented with 10% heat-inactivated fetal bovine serum (FBS; ThermoFisher Scientific Inc., Waltham, MA, USA), penicillin/streptomycin (100 U/ml and 100 μg/ml, respectively; GE Healthcare, Salt Lake City, UT, USA), 55 μM β-mercaptoethanol (Life Technologies, Carlsbad, CA, USA), and 20 ng/ml mouse GM-CSF (PeproTech, Rocky Hill, NJ, USA) in tissue-culture-treated plates. On Days 2 and 4 of culture, floating cells were removed gently, and fresh medium was added. On day 6 of culture with GM-CSF, non-adherent and loosely adherent cells were cultured in Petri dishes for 24 h to allow GM-BMDMs to adhere. Non-adherent cells were collected and used as BMDCs.

BMDMs were generated by culturing cells for 7 days in RPMI 1640 medium supplemented with 10% heat-inactivated FBS, penicillin/streptomycin, and 20% L929 conditioned medium (as a source of macrophage colony-stimulating factor)^62^. BMDCs and BMDMs were stimulated (or not) with LPS and then analyzed.

The experiments were repeated independently at least three times with at least three replicates. Each figure legend contained a sample size (*n*) for each experimental condition, given as an exact number.

### DC and T-cell co-cultures

Male C57BL/6 mice were immunized using an emulsion of chicken CII and CFA as described above. They then received a booster immunization of CII in IFA into the left hind paw on Day 8. Three days later, single-cell suspensions were prepared from iLNs by digestion with collagenase D. CD4^+^ T-cells were obtained by positive selection on CD4-Microbeads (Miltenyi Biotec, Bergisch, Germany). These cells were then labeled with 1 μM 5(6)-carboxyfluorescein diacetate succinimidyl ester (CFSE; Life Technologies, CA, USA). BMDCs from the *TonEBP*^*fl/fl*^ *LysM-cre* and littermate control *TonEBP*^*fl/fl*^ mice were cultured in 96-well plates (1 × 10^5^ per well) and stimulated with LPS (100 ng/ml) plus CII (20 μg/ml) for 24 h. Next, cells were washed thoroughly and co-cultured with the CFSE-labeled CD4^+^ T-cells (1 × 10^6^ per well) for 4 days. CD4^+^ T-cells alone without BMDCs were used as a control. Cells were harvested at 2, 3, or 4 days and analyzed by flow cytometry.

### Phenotypic analysis of intracellular cytokine and surface marker expression

Immunophenotyping of cells was performed by flow cytometry using multicolor fluorochrome-conjugated antibodies. Identification of cell surface markers was performed using antibodies purchased from BD Biosciences (San Jose, CA, USA; anti-CD11c FITC; clone HL3, anti-MHCII PE; clone 2G9, anti-CD86 APC; clone 2331 [FUN-1], anti-CD4 PerCP-Cy; clone RM4–5, anti-CD11b PerCP-Cy; clone M1/70, anti-F4/80 BV421; clone T45-2342) or BioLegend (San Diego, CA, USA; anti-CD80 PE/Cy7; clone 16-10A1) and used at a dilution of 1:100–1:500 unless stated otherwise. After surface-staining, anti-IFN-γ FITC (clone B27) and anti-IL-17 PE (clone TC11-18H10) (BD Biosciences) were used for intracellular staining using a fixation and permeabilization kit (BD Biosciences). Flow cytometry data were acquired on a BD LSR Fortessa (BD Biosciences), and data were analyzed using FlowJo software (Tree Star, Ashland, OR, USA).

### RNA isolation and reverse transcription PCR

Total RNA was extracted from paw tissue, BMDCs, and BMDMs using TRIzol reagent (Invitrogen, Carlsbad, CA, USA), and complementary DNA was synthesized using M-MLV reverse transcriptase (Promega, Madison, WI, USA). Next, real-time PCR was performed using SYBR Green I Master and a LightCycler 480 II (Roche, Rotkreuz, Switzerland). The cycling conditions were as follows: 95 °C for 5 min, followed by 45 cycles of 95 °C for 10 s, 60 °C for 15 s, and 72 °C for 20 s. Measured cycle threshold values were normalized to the cyclophilin A gene and expressed as fold changes relative to control samples. The primers are described in Supplementary Table 1.

### Immunoblot analysis

Cells were washed twice with cold PBS and lysed in RIPA buffer (0.01 M Tris, pH 7.4, 0.15 M NaCl, 0.001 M EDTA, 0.001 M EGTA, 1% Triton-X 100; all from Sigma-Aldrich) containing 0.002 M PMSF (Sigma-Aldrich) and protease inhibitors (Roche). After centrifugation of the lysate, the supernatant was used for immunoblot analysis. The protein concentration was measured using the BCA protein assay system (Pierce Biotechnology, Rockford, IL, USA). Proteins were denatured in Laemmli buffer, and equal amounts from each sample were separated on SDS–polyacrylamide gels prior to transfer to PVDF membranes. Blocking, incubation with primary antibody, and washing of the membrane were performed in PBS supplemented with 0.05% Tween-20 (v/v) and 5% (w/v) non-fat dry milk. The primary antibodies used for immunoblotting were anti-TonEBP (16; 1:3000), anti-phospho-p38 (#4511), anti-p38 (#8690), anti-phospho-Erk (#4370), anti-Erk (#9102), anti-phospho-SAPK/JNK (#4668), anti-SAPK/JNK (#9252) (all from Cell Signaling Technology), anti-CREB (sc-377154, SantaCruz Biotechnology, Santa Cruz, CA, USA), and anti-phospho-CREB (sc-7978, SantaCruz Biotechnology). Anti-Hsc70 (#200-301-A28; Rockland, Limerick, PA, USA) was used as a loading control. All antibodies were used at a 1:1000 dilution unless stated otherwise. Horseradish-peroxidase-conjugated goat anti-mouse (62-6520; ThermoFisher Scientific, Inc.) or anti-rabbit (65-6120; ThermoFisher Scientific, Inc.) secondary antibodies were diluted 1:5000. Reactive bands were detected by chemiluminescence using the ImageQuant LAS 4000 imaging system (GE healthcare).

### Cytokine analysis

Cytokine (TNF-α, IL-6, IL-17, IFN-γ, CCL-2, and IL-1β) levels in serum obtained from mice or culture medium were measured using enzyme-linked immunosorbent assay kits (R&D Systems, Minneapolis, MN, USA).

### Statistical analysis

Data are expressed as the mean ± standard error of the mean (s.e.m.) or standard deviation (s.d.). The statistical significance of differences between two conditions was estimated using an unpaired *t*‐test. One‐way ANOVA was used to compare multiple (more than two) conditions. Tukey’s post hoc test was used for multiple comparisons. A *p*-value <0.05 was deemed significant. The variance was similar between groups that were being statistically compared. All statistical analyses were performed using GraphPad Prism 8.2 software (GraphPad, San Jose, CA, USA). No data were excluded from the analyses.

## Supplementary information

Supplementary Method_Figure legends_Table

Supplementary Figure 1

Supplementary Figure 2

Supplementary Figure 3

Supplementary Figure 4

Supplementary Figure 5

Supplementary Figure 6

Supplementary Figure 1-6_merged file
