# Supplementary material for: TonEBP in dendritic cells mediates pro-inflammatory maturation and Th1/Th17 responses
Source: Cell Death Dis. 2020 Jun 4;11(6):421. doi: 10.1038/s41419-020-2632-8 (PMC7272407; doi:10.1038/s41419-020-2632-8)
Supplement: Supplementary file 1 — Supplementary Method_Figure legends_Table [file 41419_2020_2632_MOESM1_ESM.docx]

**Supplementary Information**

**TonEBP in dendritic cells mediates pro-inflammatory maturation and Th1/Th17 responses**

**Ye *et al.***

**Supplementary information**

**Supplementary Methods**

**Adjuvant-induced arthritis**

Male *TonEBP^fl/fl^ LysM-cre* mice and littermate control *TonEBP^fl/fl^* mice (aged 8–9 weeks) were immunized (by injection into the knee joint of the hind limb) with 200 μg CFA (Chondrex, Redmond, WA, USA) containing heat-killed *Mycobacterium tuberculosis*. The same amount of IFA (Chondrex) was injected into the opposite knee joint as a negative control. Paw volume was monitored every 3 days up until 14 days post-immunization using a plethysmometer.

**Peritoneal macrophage preparation**

Mouse peritonitis was induced by intraperitoneal (i.p.) injection of 3% thioglycolate broth to 8- to 10-week-old mice. Peritoneal cells were harvested at 72 h later and macrophages were enriched by quick adhesion to culture dish.

**Assays for the quantification of cell proliferation, cell viability and apoptosis**

Cell proliferation and cell viability were measured by Trypan blue staining and MTT assays. Cell apoptosis was detected with flow cytometry using an annexin V-FITC apoptosis detection kit (BD Biosciences, San Jose, CA, USA).

**Supplementary Figure 1. Myeloid TonEBP deficiency reduces the severity of arthritis.**

(**A**) TonEBP immunoblotting from peritoneal macrophages, bone marrow-derived macrophages (BMDMs), and bone marrow-derived dendritic cells (BMDCs) obtained from *TonEBP^fl/fl^ LysM-cre* and *TonEBP^fl/fl^* mice. (**B**) Study design for induction of collagen-induced arthritis (CIA) in mice. Mice were immunized with collagen type II (CII) in complete Freund’s adjuvant (CFA) (Day -14) and then were boosted 14 days (Day 0) later. Arthritic severity scores were monitored every 4th day post-booster immunization. Mice were sacrificed on day 28 after booster immunization. (**C**) Study design for adjuvant-induced arthritis (AIA) in mice. Mice were immunized with complete Freund’s adjuvant (CFA) or incomplete Freund’s adjuvant (IFA), and paw volume was monitored every 3rd day up until 14 days post-immunization. (**D**) Paw volume of AIA mice on the indicated days post-immunization (*n* = 5 per group). *n* represents the number of biologically independent animals. Data are expressed as the mean + s.e.m. # *p* < 0.05 *vs.* 0 days. * *p* < 0.05 vs. *TonEBP^fl/fl^* (unpaired t-test). (**E**) Representative images of hind paws of AIA mice on day 14 post-immunization.

**Supplementary Figure 2. Increased immune responses in paw tissue of CIA mice.**

CIA was induced in male *TonEBP^fl/fl^* mice aged 8–9 weeks (*n* = 8). Normal animals were treated with CFA (vehicle). (*n* = 8). Expression of mRNA encoding TonEBP and pro-inflammatory cytokines (**A**), matrix metalloproteinases (**B**), and Th1 and Th17 cytokines (**C**) in paw tissue was measured by quantitative RT-PCR on day 28 post-booster immunization. mRNA levels were normalized to the level of the cyclophilin A mRNA. *n* represents the number of biologically independent animals. All data are expressed as the mean + s.e.m. ND: not detected (Ct < 39). * *p* < 0.05 vs. Normal (unpaired t-test).

**Supplementary Figure 3. Flow cytometry gating strategies and the expression of DC migration-related genes in inguinal lymph nodes of CIA mice.**

CIA was induced as in Supplementary Figure 2 using *TonEBP^fl/fl^ LysM-cre* mice and their *TonEBP^fl/fl^* littermates. (A) Representative flow cytometry gating strategies of IFN-γ- and IL-17-expressing CD4+ T-cells from inguinal lymph nodes (iLNs) on day 7 post-booster immunization. (B) Representative flow cytometry gating strategies of CD11c^+^ dendritic cells within the cell population in iLNs on day 7 post-booster immunization. (C) Expression of mRNA encoding CCR7, CCL19 and CCL21 in iLNs was measured by quantitative RT-PCR on day 7 post-booster immunization. mRNA levels were normalized to the level of the cyclophilin A mRNA. Each dot is from an independent animal. Mean + s.e.m. Not significantly different (unpaired t-test).

**Supplementary Figure 4. Generation and characterization of BMDCs.**

Bone marrow-derived dendritic cells (BMDCs) were generated as described in Methods. On day 6 of culture with GM-CSF, non-adherent and loosely adherent cells were transferred to Petri dishes to remove GM-CSF grown BM-derived macrophages (GM-BMDM). After 1 day of culture, non-adherent cells (BMDC) and adherent cells (GM-BMDM) were harvested. (**A**) Expression of surface markers was analyzed by flow cytometry. Representative tracings for CD11b, CD11c, MHC II, CD86, CD80 and F4/80 **(top)**. Mean fluorescence intensity of surface markers in meand ± SD, n = 3 **(bottom)**. (**B and C**) Expression of mRNA encoding DC-related genes RelB, CCR7, CD86 and CD80 **(B)** and macrophage-related genes MafA and MafB (**C**) was measured by quantitative RT-PCR in BMDCs and GM-BMDMs. Mean ± SD, *n* = 4. *p<0.05. (**D**) BMDCs were generated from 24×10^6^ BM cells of *TonEBP^fl/fl^ LysM-cre* mice and *TonEBP^fl/fl^* littermates as above (*n* = 3). Cells numbers were counted with trypan blue staining at the indicated times **(left)**. Expression of CD11c was analyzed by flow cytometry **(right)**. **(E)** BMDCs and BMDMs were generated from *TonEBP^fl/fl^ LysM-cre* mice and *TonEBP^fl/fl^* littermates. Expression of mRNA encoding MHC II-related genes (H2-Aa, H2-Ab, and CD74) in BMDCs (**left**) and BMDMs (**right**) was measured by quantitative RT-PCR (*n* = 4). *n* represents the number of biologically independent samples. All data are expressed as the mean + s.d. * *p* < 0.05 vs. *TonEBP^fl/fl^* BMDM (one-way ANOVA).

**Supplementary Figure 5. Expression of DC maturation markers in BMDCs.**

BMDCs were generated from *TonEBP^fl/fl^ LysM-cre* mice and *TonEBP^fl/fl^* littermates and were stimulated for 24 h with phosphate-buffered saline (Veh) or LPS (100 ng/ml) (*n* = 3). **(A)** Surface markers of DC maturation (MHC II, CD86, and CD80) were analyzed by flow cytometry in BMDCs. Representative images (**top**) and quantification (**bottom**) of cell surface expression of MHC II, CD86, and CD80. (**B**) Expression of mRNA encoding TLR4 and CD14 in BMDCs. (**C**) Cell viability measured by MTT assay in BMDCs. (**D**) The percentage of apoptotic cells measured by Annexin V-staining in BMDCs. Cycloheximide (CHX) was used as positive control. Representative images (**top**) and quantification (**bottom**) of cells stained for annexin V. *n* represents the number of biologically independent samples. All data are expressed as the mean + s.d. #, * p < 0.05 vs. Veh. (one-way ANOVA).

**Supplementary Figure 6. Expression of MHC II-related genes in BMDCs and BMDMs.**

BMDCs and BMDMs generated from *TonEBP^fl/fl^ LysM-cre* mice and *TonEBP^fl/fl^* littermates were stimulated for 6 h with LPS. Expression of mRNA encoding MHC II (H2-Aa, H2-Ab, and CD74) and their transcriptional coactivator class II transactivator (CIITA) was measured in BMDCs (**A**) and BMDMs (**B**) (*n* = 3) by quantitative RT-PCR. *n* represents the number of biologically independent samples. All data are expressed as the mean + s.d. * *p* < 0.05 vs. *TonEBP^fl/fl^* BMDM (one-way ANOVA).

**Supplementary Table 1. Primers used for real time PCR**

| Species | Gene | Forward primer (5'-3') | Reverse primer (5'-3') |
| --- | --- | --- | --- |
| Mouse | *CCL19* | ATGTGAATCACTCTGGCCCAGGAA | AAGCGGCTTTATTGGAAGCTCTGC |
|  | *CCL21* | TGAGCTATGTGCAAACCCTGAGGA | TGAGGGCTGTGTCTGTTCAGTTCT |
|  | *CCR7* | TCATTGCCGTGGTGGTAGTCTTCA | ATGTTGAGCTGCTTGCTGGTTTCG |
|  | *CD14* | GGAAGCCAGAGAACACCATC | CCAGAAGCAACAGCAACAAG |
|  | *CD74* | TTGCTGATGCGTCCAATGTC | GGGTCATGTTGCCGTACTTG |
|  | *CD80* | GAGGCAAGCAGAGAAACAAAC | GTATCCCACATGGACAGAGAAG |
|  | *CD86* | TCACCCGAAACCTAAGAAGATG | AGAGAGAGGCTGTTGGAGATA |
|  | *CIIA* | AGGCCTATGCCAACATTGCG | CCATAGCATGCTCTTCCGGG |
|  | *H2-Aa* | AGGTGAAGACGACATTGAGG | AACTCAGGAAGCATCCAGAC |
|  | *H2-Ab* | CCATTACCTGTGCCTTAGAG | GAACTGGTACACGAAATGCC |
|  | *IFNg* | TGAGCTCATTGAATGCTTGG | ACAGCAAGGCGAAAAAGGAT |
|  | *IL-12p40* | TGGTTTGCCATCGTTTTGCTG | ACAGGTGAGGTTCACTGTTTCT |
|  | *IL-17* | TCCAGAATGTGAAGGTCAACC | TATCAGGGTCTTCATTGCGG |
|  | *IL-18* | CAGCCTGTGTTCGAGGATATG | TCACAGCCAGTCCTCTTACT |
|  | *IL-1b* | ATACTGCCTGCCTGAAGCTCTTGT | AAGGGCTGCTTCCAAACCTTTGAC |
|  | *IL-2* | ATGTACAGCATGCAGCTCGCATC | GGCTTGTTGAGATGATGCTTTGACA |
|  | *IL-21* | GGAGACTCAGTTCTGGTGGC | GAGCGTCTATAGTGTCCGGC |
|  | *IL-23* | AGATAACAACACAGATGTCC | CTTGATCTCTCCACTCTCTC |
|  | *IL-6* | ATCCAGTTGCCTTCTTGGGACTGA | TAAGCCTCCGACTTGTGAAGTGGT |
|  | *MafA* | AGCAGTTGGTGACCATGTCG | TGGAGATCTCCTGCTTGAGG |
|  | *MafB* | CCATCTTGAGAAGGTAGCAGCAA | AAAGTTGGGCTTGGTGGGTT |
|  | *MCP-1* | AACTGCATCTGCCCTAAGGT | AGTGCTTGAGGTGGTTGTGGAA |
|  | *MMP-13* | GGT CCT TGG AGT GAT CCA GA | TGA TGA AAC CTG GAC AAG CA |
|  | *MMP-2* | ACCAAGAACTTCCGATTATCCC | CAGTACCAGTGTCAGTATCAGC |
|  | *MMP-9* | GATCCCCAGAGCGTCATTC | CCACCTTGTTCACCTCATTTTG |
|  | *RelB* | CCGTACCTGGTCATCACAGAG | CAGTCTCGAAGCTCGATGGC |
|  | *TGF-b* | ATTTGGAGCCTGGACACACAGT | TTGCAGGAGCGCACAATCATGT |
|  | *TLR4* | TGCTGCCAACATCATCCAGGAA | AGGCGATACAATTCCACCTGCT |
|  | *TonEBP* | AAGCAGCCACCACCAAACATGA | AAATTGCATGGGCTGCTGCT |
