# Supplementary material for: TonEBP in dendritic cells mediates pro-inflammatory maturation and Th1/Th17 responses
Source: Cell Death Dis. 2020 Jun 4;11(6):421. doi: 10.1038/s41419-020-2632-8 (PMC7272407; doi:10.1038/s41419-020-2632-8)

**Supplementary Figure 1**

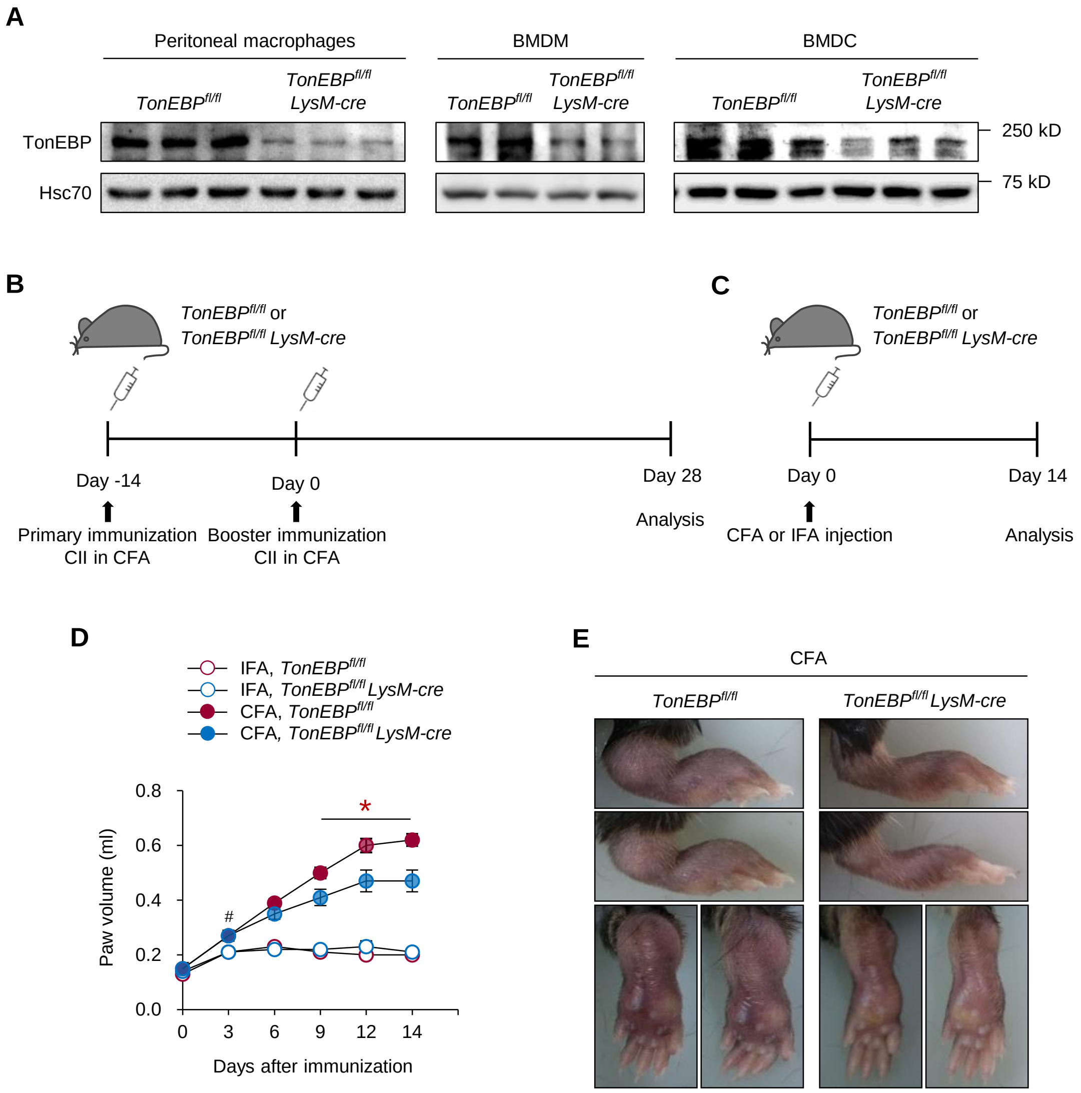

**Supplementary Figure 2**

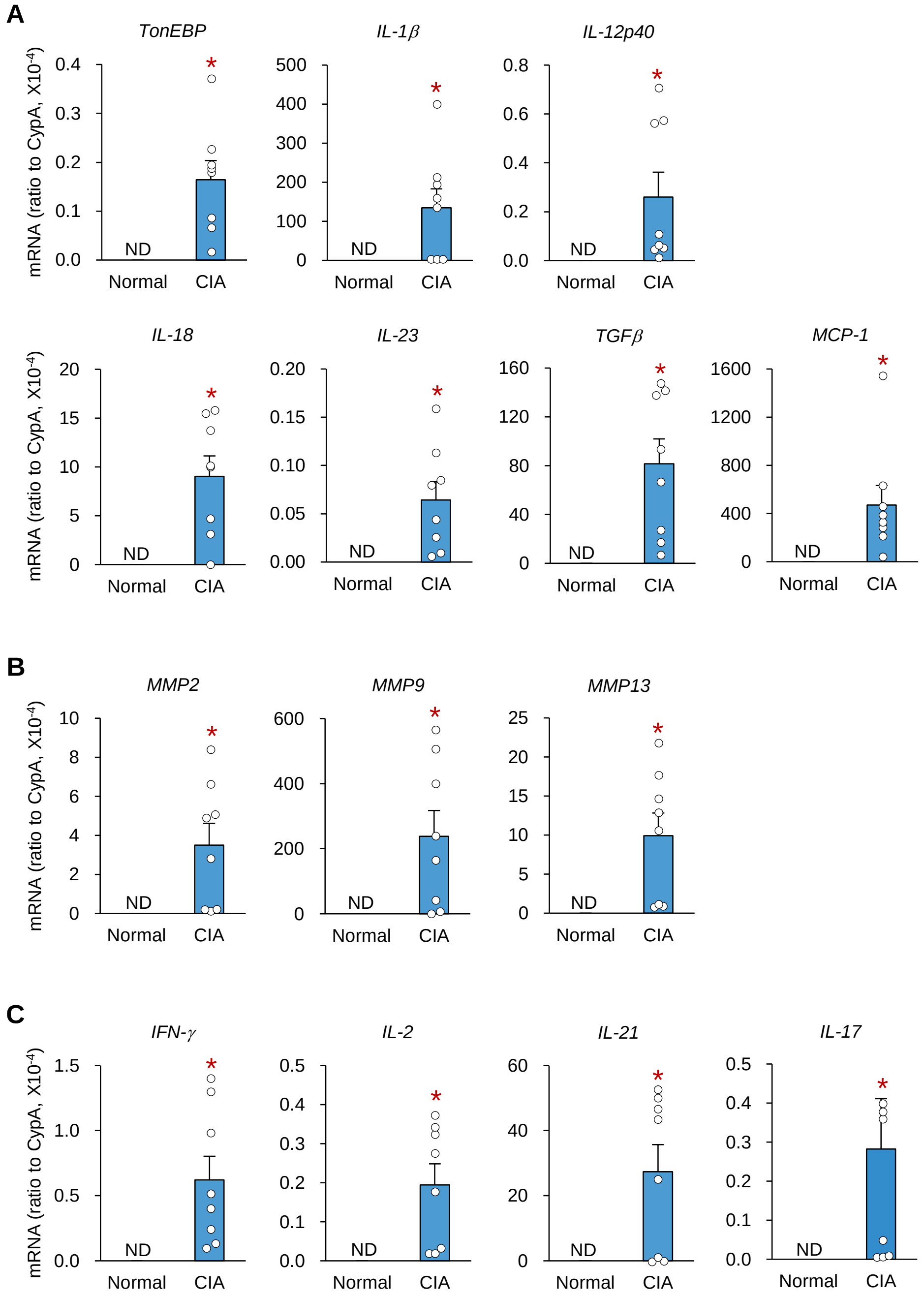

## Supplementary Figure 3

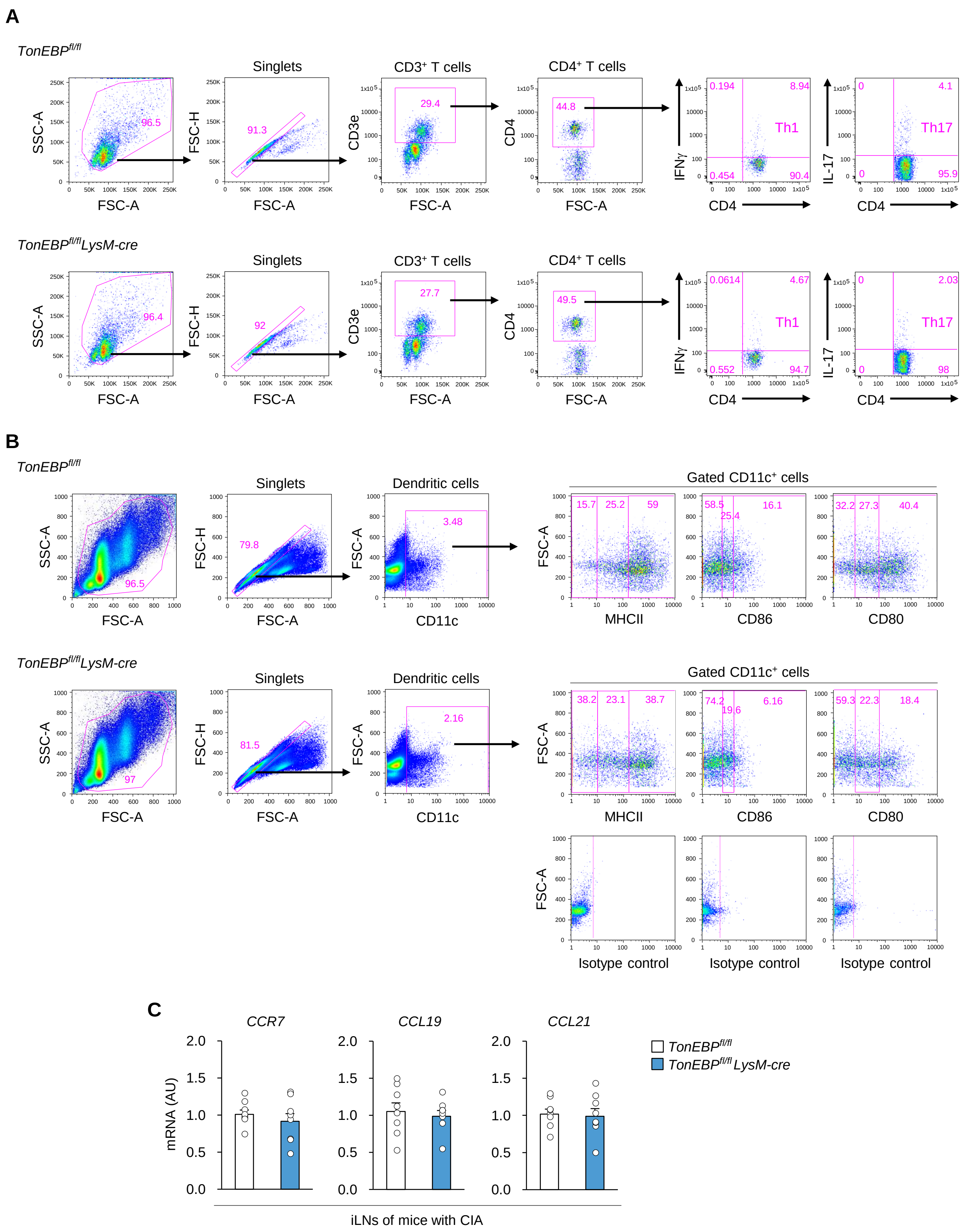

## Supplementary Figure 4

A

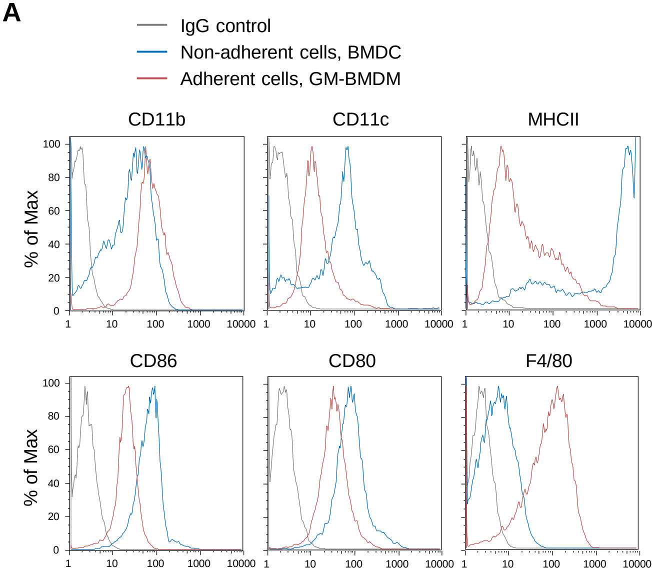


| Surface marker | Non-adherent cells, BMDC | Adherent cells, GM-BMDM |
|---|---|---|
| CD11c | 108.0 ± 5.3 | 21.2 ± 2.5 |
| MHCII | 3672.7 ± 82.7 | 103.0 ± 38.1 |
| CD86 | 110.5 ± 40.3 | 22.9 ± 1.9 |
| CD80 | 149.3 ± 44.1 | 49.1 ± 5.2 |
| F4/80 | 5.5 ± 0.8 | 132.3 ± 22.0 |

B

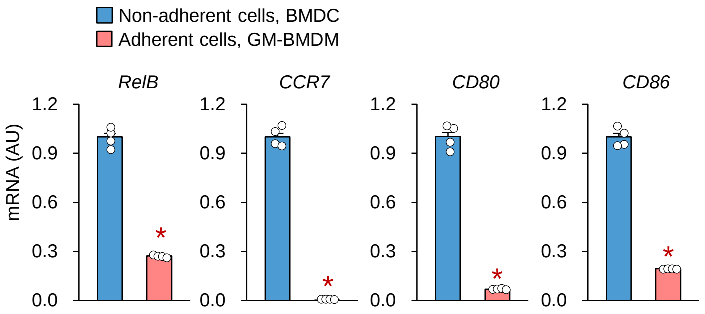


C

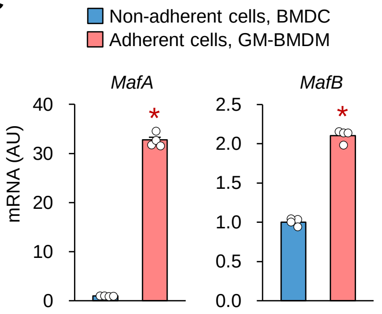


D

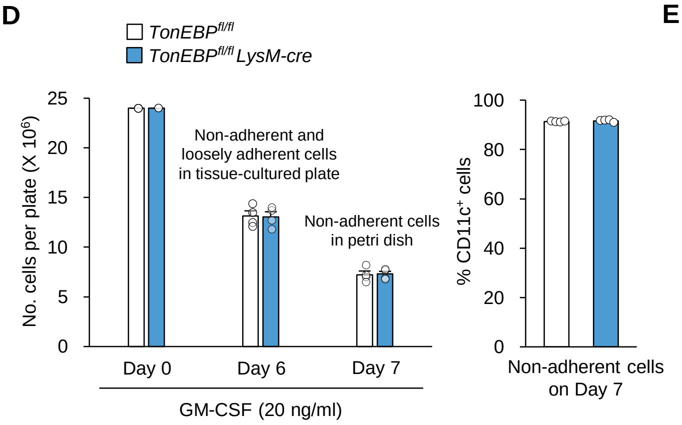


E

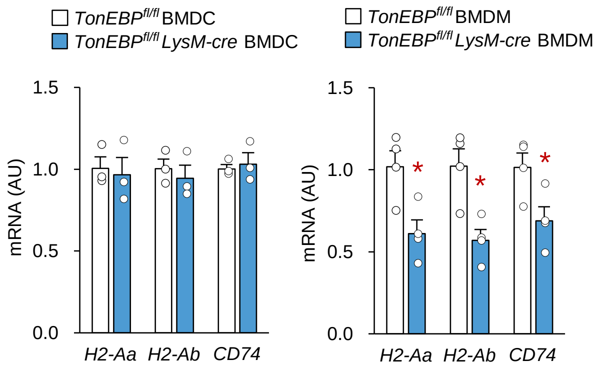

## Supplementary Figure 5

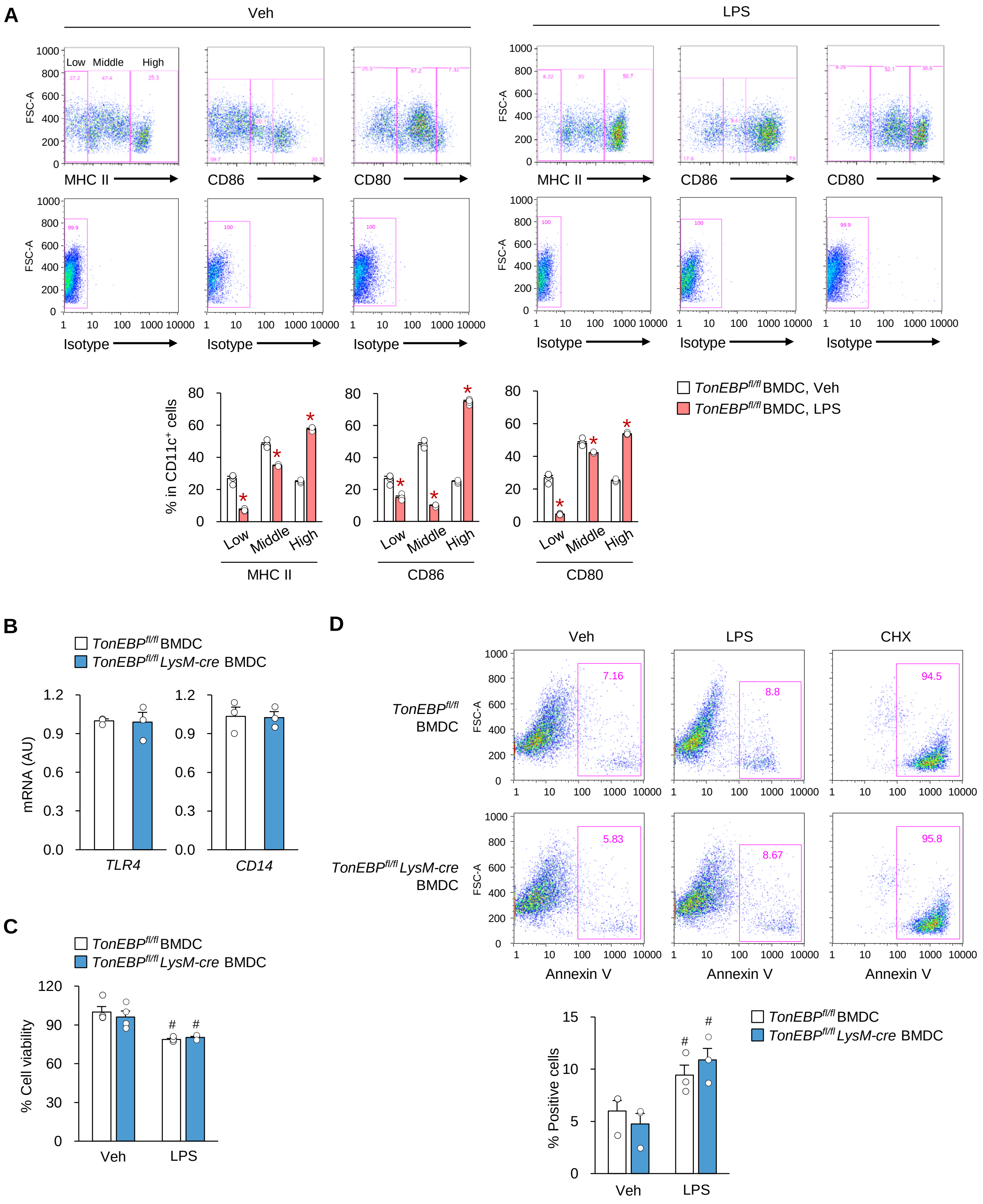

## Supplementary Figure 6

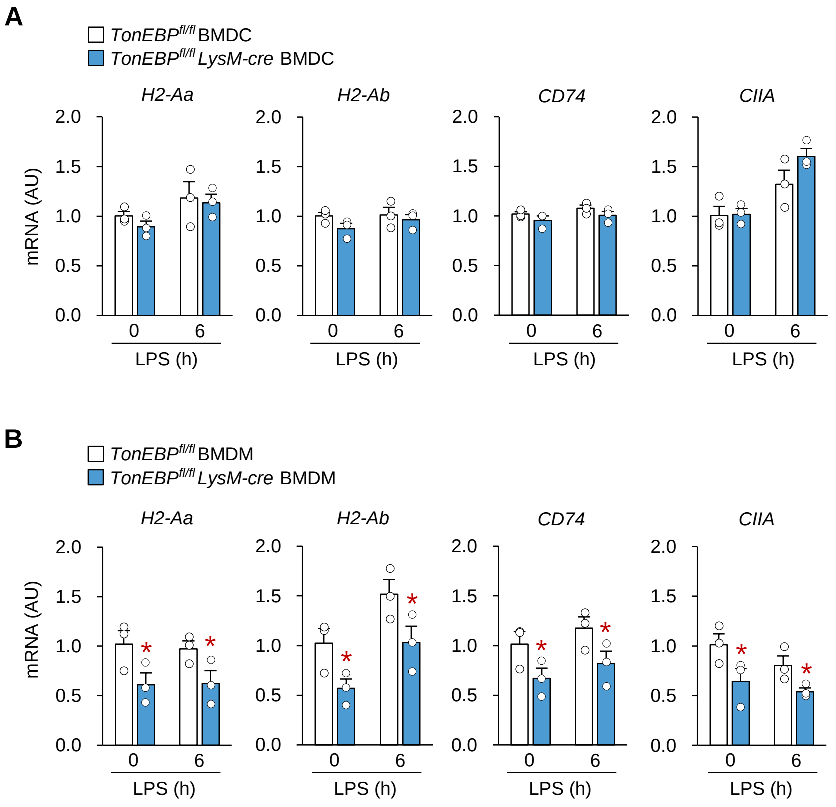

Supplement: Supplementary file 8 — Supplementary Figure 1-6_merged file [file 41419_2020_2632_MOESM8_ESM.pdf]
